# Report of a Case of Carcinoma of the Stomach

**Published:** 1851-08

**Authors:** P. H. Strong


ART. IV.—*Report of a case of Carcinoma of the Stomach.* By P. H. STRONG, M. D., read at the meeting of the Buffalo City Medical Association, July 1, 1851.

Friday, January 17, 1851, I was called to see C. S. B., aged about 40, constitution unimpaired by previous disease, never before having required a professional visit within his recollection. Temperament, somewhere betwixt sanguine and bilious.

He had, he said, refrained from business some three weeks by constraint, feeling unable to be relied on for business, though all the while able to be walking, sitting, or lounging about the public rooms of his hotel. He traced the first accession of his symptoms back some two or three months, and supposed for the most part they were *dyspeptic* indications, which, whenever he had leisure, and chose to devote himself to it, might be readily removed.

Feeling but slight pain, aud *that* at irregular intervals, and moreover having a good appetite and fair digestion, with regular action of the bowels, he had thought his symptoms unworthy of serious attention.*

* Upon reflection, I remembered that he had, some month or two previous, asked me, quite casually and when he had called for other ends, to give him a prescription for worms, as "he had pretty much concluded he was troubled with them." I then in-

From the time that he had quit his business, and indeed for some time previously (as I afterwards learnt from others) he had been tampering with this thing and that, according to varying fancies and feelings, as to what ailed m. He had had (I discovered) *Botanic* predilections, and under their influence had foresworn *mineral medicines*, and been in the habit of using largely of "*hot drops*," &c. This, one who knew afterwards assured me, had been an inveterate practice with him for a number of years.

*Now*, however, he "had concluded he did not understand his case," had become somewhat alarmed about himself, and was prepared to submit to anything I thought best.

It should be farther premised, that he had, for a few weeks previous to my visit, occasional, though irregular, *vomitings*. These occurred generally from three to five or six hours after eating, when he had felt his desire for food renewed, and perhaps was on his way for its gratification — for the most part once a day, and almost uniformly, as he reported, consisted of "*pure yellow bile*," with no admixture of the *ingesta* — occurring thus, apparently, upon a stomach *emptied of food*.

It should farther be added, that he craved the most concentrated diet, and was in the habit of taking largely, and without restriction, the strongest and most indigestible meats and vegetables, and that, too, strange as it may seem in the sequel, without experiencing appreciable inconvenience.

So much for the history of the case as gathered from the patient and friends.

At my first visit, my patient was about the public rooms of the hotel, satisfied that he was sick beyond his own skill to remove, but not apprehensive of the serious character of his case.

His pulse was about eighty. Respiration normal — muscular energy not greatly impaired — slightly emaciated — appetite good — tongue loaded with a thick *fur-like* coat — bowel and urinary excretions not essentially unnatural — countenance yellow and somewhat hippocratic — skin perspirable — night rest pretty good, except as "the pain in his side," as he termed it, hindered.

To *this*, upon exploration of his abdomen, my attention was immediately drawn. There was great tenderness upon pressure, *at*, and directly below

---

quired what reason he had for his opinion, and upon his report of his symptoms and feelings, I remember of thinking that he *might* be thus troubled, but that his symptoms more indicated *chronic colonitis*. This was quite casual and unimportant, and I refer to it now as not being at that time impressed that he had any serious, at least, *malignant* disease, from his general appearance, and his own report.

and at the left of the *pit of the stomach*, and all through the *left hypochondrium*. But what most attracted my attention, and impressed me as the seat of his disease, was a *distinct*, *slightly moveable*, though *unimpressible tumor*, of about the size of my fist, situate near, yet *below*, and slightly at the left of the stomach pit, and extending indefinitely back. The pain, on pressure, was not so much referred to the *tumor* as to points at the left, and under the false ribs.

I at once called his attention to the *tumor*, and *its existence seemed to take him quite by surprise*. He had not before noticed any tumefaction. I told him that the *original* and *real* trouble, to my mind, was in the *tumor*, though he referred his distress to, and still seemed to think the difficulty more to the left, and under the false ribs.

As to its *nature* and *seat*, my mind vibrated for two or three days, between enlarged and displaced *spleen* and *scirrhus* of the *pyloric extremity* of the *stomach*, with *colonitic* inflammation and attachments. I did not, at that time, nor for several days, express to him my full apprehensions as to the nature of the disease, in the hope, that by reducing the evident inflammation in the parts contiguous, and correcting the stomach derangement, his case might present somewhat more hopefully. A very free application of leeches for a day or two, conjoined with four or five five grain doses of calomel, and succeeded by a blister, met my most sanguine expectations, so far as removing the inflammatory symptoms was concerned, and procured characteristic fecal discharges, but left the coat on the tongue unaffected.

The course thus sketched, with morphine to procure night rest and sleep, relieved him almost wholly, and kindled up a fresh courage and confidence of recovery within him. He frequently remarked that he was sure he was better, "that he felt the disease was fast being overcome." He "could *feel* it giving way," &c.

Not *so*, however, was I impressed — my worst fears began to assume shape and form. The *tumor* remained essentially unchanged, and I found my mind fast settling upon its *scirrhus-carcinomatous* character, and *that*, as I thought, of the *pyloric extremity of the stomach*. I requested a consultation, and *Dr. Peabody*, of this city, was called, who concurred in its being of a malignant, and probably fatal character, though, as we both thought, with little likelihood of a speedy termination. Indeed there seemed, from the slight inroad the disease had yet made upon his vital energies, a possibility that he might yet be about, perhaps for months. As to treatment, too, there was substantial agreement. But I need not take time to detail the daily progress of the case.

From this time (about the 23d of Jan.) till the middle of Feb., about three weeks, there was no marked *change*, nor *any* features of the case worth reporting, except, indeed, it be his unlimited confidence of restoration to health. And that, too, notwithstanding my uniformly unfavorable prognosis, expressed with even unwonted plainness. This was accounted for, measurably, by the complete relief from pain and wakefulness, and by the lengthened interval between vomiting that he had gained, and, perhaps may be added, his sustained relish for food, and the slight failure of vital and muscular energies and strength. To whatever attributable, however, I have never seen in a patient so great, I may say, *extravagant* courage and confidence of recovery, at the very time that I was assuring him that he was "hoping against hope," there being little or nothing to base a hope upon. He seemed lit up with hope and assurance of recovery, in an inverse ratio to the chance of such an issue. Remarking that "though he believed we were right in the main, he thought we had underrated the wonderful energies of his system."

He continued much the same until about the 17th of Feb., when, becoming impressed that his disease was insidiously preying upon the energies of his system (though he was yet sure he was improving) I requested another consultation, and *Dr. Flint* was summoned. Upon examination, he thought there could be little or no doubt as to the correctness of the *diagnosis*. He, too, could not see why he might not hold out for weeks, perhaps months. Judge my surprise, when, upon the next day, before the hour set for my visit, I received notice that my patient was thought to be dying! I hastened to him, but before I could reach his bedside, life was extinct.

It appeared that he had felt as well as usual through the forenoon of the day, ate his dinner with his usual relish, and, whilst his attendant was at dinner, had got up alone and helped himself, at the operation of some *castor oil*, that had been given, and on his return to bed was taken with fainting, was partially restored, again relapsed, and was soon seen to be "passing away." His death in its suddenness, took all by surprise, in which I fully shared, and even now, after much reflection, I confess myself unable to account for it satisfactorily.

Further detail of treatment has been purposely omitted till now, with a view to economy of space and time. It may be said to have consisted, after what was first specified, and whilst there was the least possible doubt of the *nature* of the disease, of a free use of *Pot-Hyd-Iod.* internally, and *Tinc-iod.* externally, with occasional mercurials to restrain distressing vomitings. He was kept altogether comfortable by the regular use of *morphine* througho his sickness. Of course nothing that was done availed to the slightest extent toward *removing* his *disease.* All the good that it is possible to say of it is, that it *may* have postponed the event for a few *days,* possibly *weeks.*

*Autops-cadav.* 24 hours after death. Present — Drs. Flint, Samo, Ford, Hawley, Gray, Jewett, Eastman, T. D. Strong.

Search being directly made for the tumor, it was found to be the *stomach.* Our diagnosis was at once confirmed as to its *nature,* (*carcinoma,*) and, *measurably,* as to its *seat* and *extent.* The *lower,* or *pyloric third* of the organ, however, and the upper curvature, were the least involved, while the *pyloric valve* and the *duodenum* (the most suspected portions) were quite free from disease. Its capacity was rated at not over one pint, and quite insusceptible of distension. Its whole structure was changed, except it be portions of the mucous coat. There was little resemblance to a *serous,* and none at all to a *muscular coat,* both being degenerated into scirrhus of almost cartilaginous hardness, responding to the scalpel somewhat like *half-tanned bulls hide,* though of harder and less fibrous structure. The cut surface resembled very much in its color and look, a *broken* surface of *new rich cheese.* The mucous coat was found the least altered in its structure, a good deal of its surface appearing no worse than, and much like, that of the victim of *delirium tremens.* There were ulcerated spots of from half-inch to two inches diameter. One or two of which had eaten nearly through the transformed tissues, where they were toward an inch thick, and were of a most fetid cancerous degeneration.

The parts generally of most thickness and hardness, were throughout the *greater curvature,* and toward the *cardiae orifice.* The point that presented to constitute the tumor externally, was the lower and anterior surface, about one-third of its length from the *pyloric orifice.*

The contiguous colon was adherent to the stomach, and at its adhering surface was considerably involved in the carcinomatous degeneration. There were found, too, several of the contiguous *mesenteric glands* of varying sizes, the maximum being (say) three-fourths of an inch in diameter, whose structure was entirely transformed by the *carcinomatous deposit.*

The remaining *abdominal viscera,* (with the exception of a few *tubercles* on the upper surface of the *liver*) as also the *heart* and *lungs,* were found healthy. Brain not examined.

Our surprise at the suddenness of the termination of the case, gave place, at once, on seeing the terrible malignancy and extent of the disease of the stomach, to astonishment that life had been maintained *at all* for weeks, and perhaps months ; for it would seem that the great extent, as well as the peculiar nature of the disorganization, would preclude the supposition that the disease could have been less than one or two years (probably *more*) in progress.

One or two po[illegible] i[illegible] h[illegible] case seem deserving of especial notice, with a view to elucidation, and to being fixed in the memory.

1st. Its insidiousness, and the utter lack of urgent symptoms, until within a *few weeks of its termination.* At *no* time could any *adequate* idea of his disease have been impressed upon the mind of the patient, *even had it been suspected, which* in its extent, I freely admit it was not. But there was hardly sufficient to attract his *slightest* notice till within two or three months of his death. Most evidently the disease must have made serious inroads upon his stomach long enough before *this.*

2d. The almost *unimpaired digestion* that terminated only with his life. It is not unreasonable to suppose that there have been other cases, in which the whole stomach was involved, and, for the most part, disorganized. But, certainly not, from quite an extensive examination of the history of the disease, and all the records of cases, to which your reporter has had access, could I have inferred, or perhaps have been *justified in diagnosing so extensive, and prognosing so speedily fatal* a malady as this proved.

> "The times have been
> "That, when the (*stomach*) was out, the *man* would *die,*
> "And there an end."

The present case points to a modification of our former views in this regard, for it would really seem as good as "*out*" in its capacity of a *stomach.* But seriously—

Quere 1st. Where was digestion carried on? For it will be remembered, *that* function was nearly unimpaired as indicated by exemption from uneasiness after taking hearty food, as also by, in the main, quite well formed feces, with natural and easy defecation. Was it by the mucous coat, wholly disorganized and destroyed in *much*, and greatly diseased in nearly the *whole* of its extent, with but slight portions, *and those upon its upper surface,* of anything approximating to healthy mucous membrane — or did the duodenum assume the function? The uniform absence of anything that had been eaten in what was vomited, would indicate the latter as the true explanation.

Quere 2d. How much (if any) of the exemption from pain in the stomach is referrible to the wholly degenerated muscular coat and the consequent insusceptibility of distension?

Quere 3d. How account for the ejections from the stomach, with the absence of the muscular coat, or the trifling amount remaining near its orifices?

Other points, such as the extreme suddenness of the termination, with hardly perceptible deterioriation of vital energy for days previous, it would be agreeable to the present reporter to have cleared up by the gentlemen of the Association, as also any other suggestions that time and inclination may allow.

Perhaps I should not omit to mention as having etiological bearings, that my patient, as your reporter subsequently learned, was the subject, at the time of his death, of great anxiety and depression of spirits, from a domestic infelicity which had existed for two or three years.

